# Health, health behaviors, and health dissimilarities predict divorce: results from the HUNT study

**DOI:** 10.1186/s40359-015-0072-5

**Published:** 2015-05-01

**Authors:** Fartein Ask Torvik, Kristin Gustavson, Espen Røysamb, Kristian Tambs

**Affiliations:** Division of Mental Health, Norwegian Institute of Public Health, Postbox 4404, Nydalen, 0403 Oslo, Norway; Department of Psychology, University of Oslo, Postbox 1049, Blindern, 0317 Oslo, Norway

**Keywords:** Divorce, Marital dissolution, Spousal similarity, Assortative mating

## Abstract

**Background:**

Poor health and health behaviors are associated with divorce. This study investigates the degree to which six health indicators and health behaviors among husbands and wives are prospectively related to divorce, and whether spousal similarities in these factors are related to a reduced risk of marital dissolution. Theoretically, a reduced risk is possible, because spousal similarity can help the couple’s adaptive processes.

**Methods:**

The data come from a general population sample (19,827 couples) and 15 years of follow-up data on marital dissolution. The following characteristics were investigated: Poor subjective health, obesity, heavy drinking, mental distress, lack of exercise, and smoking. Associations between these characteristics among husbands and wives and later divorce were investigated with Cox proportional hazards regression analyses.

**Results:**

All the investigated characteristics except obesity were associated with marital dissolution. Moreover, spousal similarities in four of these characteristics (heavy drinking, mental distress, no exercise, and smoking) reduced the risk of divorce, compared to the combined main effects of husbands and wives. Nevertheless, couples concordant in these health issues still had higher risks of divorce than couples without these characteristics.

**Conclusion:**

Couples with similar health and health behavior are at a lower risk of divorce than are couples who are dissimilar in health. Health differences may thus be seen as vulnerabilities or stressors, supporting a health mismatch hypothesis. This study demonstrates that people who are similar to each other are more likely to stay together. Harmonizing partners’ health behaviors may be a target in divorce prevention.

## Background

In recent years, factors on a couple level, rather than on an individual level have received increasing attention in research on marital quality (Gonzaga et al. 2007; Dyrenforth et al. 2010). People are in general attracted to partners that are similar to themselves, and some studies indicate that spouses that are similar to each other experience higher marital satisfaction (Gonzaga et al. 2010). However, the beneficial effects of spousal similarity may not necessarily apply when the spouses are similar in characteristics that are known to be risk factors for divorce. Poor health and health related lifestyles are among the risk factors for divorce (Amato & James 2010). This study uses a prospective design to investigate several indicators of poor health and health behaviors, as well as spousal similarity in these characteristics, as risk factors for marital dissolution.

Spouses are known to resemble each other on a range of traits, including lifestyle and attitudes (Hatemi et al. 2010), mental disorders (Joutsenniemi et al. 2011), alcohol use (Reynolds et al. 2006), smoking (Reynolds et al. 2006), body mass (Meltzer et al. 2011), and exercise (Jurj et al. 2006), in addition to demographic factors (Watson et al. 2004). This similarity between spouses may be a result of initial similarity due to assortative mating (people are attracted to partners that are similar to themselves) or social homogamy (people meet partners within their own social strata). Alternatively, the similarities could arise during the marriage, either as selection effect in which dissimilar partners divorce, or through convergence (Gonzaga et al. 2010).

Spouses that resemble each other tend to have higher marital satisfaction. This has been found for values (Luo et al. 2008), alcohol use (Homish & Leonard 2005), emotions and interests (Gonzaga et al. 2010), and personality (Gonzaga et al. 2007; Luo et al. 2008; Gonzaga et al. 2010). Accordingly, the risk of marital dissolution may also be reduced when the spouses resemble each other; at least this has been found for education, attitudes, values, and life goals (Clarkwest 2007; Becker 2013), although the effects of demographic variables, such as income and education may depend on gender (Tzeng & Mare 1995; Jalovaara 2003). Among divorced people incompatibility is the second most frequently reported reason for divorce (Amato & Previti 2003).

Poor health is one risk factor for marital dissolution: Divorced individuals exhibit poorer health across a range of outcomes, such as physical health problems, mortality, cancer, suicide, smoking, alcohol use, and depression (Amato & James 2010; Dupre et al. 2009). While some of this association is likely to reflect consequences of divorce, at least a part of it is due to higher divorce rates among individuals with poor health (Bronselaer et al. 2008; Amato & James 2010).

According to the vulnerability-stress-adaption (VSA) model (Karney & Bradbury 1995), marital satisfaction depends on *enduring vulnerabilities*, which are stable characteristics that spouses bring to the marriage, and *stressful events*, which are experienced together by the spouses. The enduring vulnerabilities and stressful events have their effect on marital satisfaction through the couple’s *adaptive process*, that is, the way in which the couple cope with conflicts and marital difficulties, and how the spouses behave and feel towards each other.

Whereas spousal similarities in characteristics such as personality traits (Gonzaga et al. 2007; Luo et al. 2008; Gonzaga et al. 2010) and values (Luo et al. 2008) are related to marital functioning, as discussed above, it is uncertain how spousal similarities in risk factors for divorce, such as poor health and health behaviors relate to divorce. One may expect that experiencing malignant factors in double doses additively stress the couple and lead to a higher risk of divorce (Butterworth & Rodgers 2008). On the other hand, it is conceivable that spousal similarities even in risk factors can help the adaptive processes: Gonzaga et al. (2007) extended the VSA by looking at how spousal similarities in personality enhance positive emotional experiences as an adaptive process. According to Gonzaga et al. (2007) spousal similarity increases relationship satisfaction because similar partners share emotional experiences. They will have similar reactions to the environment, understand each other’s emotional states, and feel validated because their partner understands their feelings and behaviors. Thus, both partners may feel lonely and misunderstood if they differ in health status or lead different lifestyles. The feeling of cohesion and belonging may be reduced in such couples, whereas two partners with the same kind of challenges may be more able to support each other. Finding commonalities with partners may even be intrinsically rewarding (Becker 2013). Thus, it is possible that spousal similarities also in risk factors predict a reduced risk of divorce. Wilson and Waddoups (2002) found partial support for this “health mismatch hypothesis”. However, their health predictor was limited to a single question on subjective health, and the sample only consisted of people between 51 and 61 years of age.

In previous papers, we have investigated the specific associations between alcohol use and marital dissolution (Torvik et al. 2013), and mental distress and marital dissolution (Idstad et al. 2015). In the present paper, we aim to investigate the effects of differences in health on divorce across a broader range of health indicators and health behaviors, in models where the effects of these predictors are adjusted for each other. The current study builds on and will expand previous research by investigating a set of health variables that are possible predictors of marital dissolution: poor subjective health, obesity, heavy drinking, mental distress, lack of exercise, and smoking. These characteristics will be investigated at the couple level, estimating the effect of spousal similarity in these factors. The overarching question in this paper is thus to which extent spousal similarity, observed as the difference in all these indicators, protects against risk of divorce. Since divorce is also dependent on demographic factors (Amato & James 2010), we will adjust for age, income, education, and length of the marriage. We will use prospective data from a large general population study, with self-reported data from both spouses. Our outcome measure is marital dissolution, as recorded in governmental registries.

## Methods

### Sample and design

All inhabitants of Nord-Trøndelag County, Norway, were invited to take part in the Nord-Trøndelag Health Study (HUNT) between 1984 and 1986. The health study consisted of three parts: Invitees received a questionnaire along with the invitation letter and completed the questionnaire at home. Participants then attended a health examination. At the examination site, they returned the first questionnaire and received a second questionnaire, which was brought home and returned by prepaid mail.

The total number of invitees was 85,427, of which 64.9% were married. Individual response rates were 90.4% for the first questionnaire and 74.9% for both questionnaires, but higher among married persons. Among the invited persons, we could identify 27,307 heterosexual couples that were registered as married at the time of the survey. In 25,265 couples (92.5%) both spouses returned the first questionnaire. Both spouses returned both questionnaires in 19,977 couples (79.1%). However, 150 couples were excluded due to inconsistencies in the registries, that is, one of the spouses was registered as married, whereas the other was not. Thus, the net sample of this study consists of 19,827 couples. The average age of husbands participating in the study was 52.1 years (SD = 15.3), whereas the wives’ ages averaged 48.9 years (SD = 15.0).

The questionnaires contained a range of health and health behavior measures. In addition, demographic data from governmental registries were available, and were linked to the survey data. The participants could be followed in the governmental registries until year 2000, which gives a follow-up time of approximately 15 years. More details on the HUNT study are available at the HUNT website: www.ntnu.edu/hunt.

### Ethics

The data matching between questionnaire data and registry data and between spouses was carried out by the governmental agency Statistics Norway, using personal birth identity numbers assigned to every Norwegian citizen. All person-identifiable data were deleted before the data were returned to the researchers. Consent was granted via the return of a completed questionnaire. The Norwegian Data Inspectorate and the Regional Ethics Committee approved the study.

### Measures

#### Marriage and marital dissolution

Statistics Norway provided annual information on marital status and on who was married to whom from 1974 to 2000. This made it possible to link data between spouses and to see whether and when they divorced. Separation is treated equal to divorce, because separation is usually a stage in the divorce process. Year of marriage was inferred from the annual information on marital status for each respondent. This was combined with census data of year of marriage among the older cohorts.

#### Subjective health

Subjective health was measured with a single item (“How is your health at the moment?”). People who answered “poor” or “not so good” were coded as having poor subjective health, whereas responders answering “good” or “very good” were coded as having good subjective health.

#### Mental distress

Mental distress was measured with an anxiety and depression index (ADI-12), which consists of 12 items. These items have been weighted by coefficients calculated by Tambs and Moum (1993) to optimize the correlation (r = 0.82) between a weighted sum of these items and the Hopkins Symptom Checklist-25 (SCL-25) (Winokur et al. 1984). The ADI-12 had a theta reliability of 0.83 (Tambs & Moum 1993). The three highest loadings were on the items “Over the last month, have you suffered from nervousness (irritability, anxiety, tension or restlessness)?”, “Do you mostly feel strong and fit, or tired and worn out?”, and “Do you often feel lonely?” Responders with scores above the 90th percentile counted in all HUNT responders (not only married couples) were categorized as having clinical levels of mental distress.

#### Body mass

Body mass index (BMI) was calculated from height and weight measured at the health examination. Individuals with an BMI above 30 kg/m^2^ were defined as obese, in accordance with the World Health Organization BMI classification (The World Health Organization 2013).

#### Smoking

Participants reported whether they were daily smokers (yes/no).

#### Alcohol use

The alcohol consumption index was based on three questions – one on frequency of drinking, and two indicating hazardous drinking: “How often did you drink alcohol over the last 14 days?” (total abstainer, 0 times, 1–4 times, 5–10 times, 10 times or more), “If you drank alcohol during the past 14 days, did it make you feel influenced by alcohol on any occasion?”, (no, yes), and “Have there been periods in your life during which you have drunk excessively or at least a bit too much?” (no, not sure, yes). A few illogical responses, such as claiming to have been drunk without drinking alcohol, were set to missing. Responders were coded as “heavy drinkers” if they either drank more than 5 (women) or 10 (men) times during the last 2 weeks and admitted to 1 of the indications of hazardous drinking, or if they had both indications and had been drinking during the last 2 weeks. Different cut-off values were used for men and women because men on average drink more than women and have a higher tolerance for alcohol (Becker & Hu 2008).

#### Exercise

Frequency of physical activity was measured with one question (“By exercise we mean going for walks, skiing, swimming and working out/sports. / How often do you exercise?”). Responders who exercised once a week or more answered additional questions on exercise. We coded responders who answered “Never” or “Less than once a week” into a no exercise group.

##### Socioeconomic control variables

Age, income and highest completed education for both partners were available from the governmental registries. Age and income were used as continuously distributed variables, whereas educational attainment was scored as an ordinal variable with four levels. Income at baseline was missing for 0.02% of the responders, and education at baseline was missing for 1.3% of the participants. For these responders, income or education in 1980 or 1990 was used instead.

### Missing data

The data from governmental registries had no missing values after being treated as described above. On the other variables, missingness ranged between 0.2% (subjective health among men) and 3.1% (exercise among women). Most couples had complete data (89.0%), whereas 7.4% of couples missed one variable, 2.3% missed two variables, and 1.3% missed more than two variables. In total, 0.9% of all values in the dataset were missing due to item non-response on the questionnaires. Multiple imputation (MI) with 50 repetitions was applied to avoid excluding couples with partial responses. MI produces multiple copies of the dataset, each with random variation around the maximum likelihood estimate; thereby avoiding deflation of the standard errors. MI provides more accurate estimates than listwise deletion (Graham 2009), because it allows preserving all valid data, and, unlike imputation by expectation-maximization, MI does not deflate standard errors.

### Statistical analyses

The risk of divorce was investigated by survival analysis (Cox proportional hazard models) with risk estimates reported as hazard ratios (HR). The duration of the marriage was set as the time variable, while length of marriage at the observation was controlled for. Observations were censored either in year 2001, or when either of the spouses died.

Age is strongly associated with divorce and with most of the other characteristics. Therefore, results unadjusted for age are not informative on the other associations. Preliminary analyses showed that the results on the health and health behavior variables were approximately the same whether they were adjusted for income and education or not. Thus, all regression results are adjusted for age, income and education, in addition to marital duration.

One health indicator at a time was entered into the regression model, together with the demographic control variables. Thereafter, all the indicators were entered into the model simultaneously, in order to provide fully adjusted results.

Each of the health and health behavior variables was rendered as three variables at the couple level: the husband’s score, the wife’s score, and the interaction term between the spouses’ scores. As the variables were dichotomous, the interaction term expresses the risk of divorce when both spouses have a characteristic compared to the product of the main effects. Couples where both spouses have a characteristic can be compared to couples where none of the spouses have the characteristic by multiplying these three HRs. There are two types of concordant couples (both or no partners have a characteristic) and two types of discordant couples (either the husband or the wife has a characteristic). In order to compare concordance to discordance, we ran separate analyses in which the two concordant groups were compared to discordant couples.

## Results

### Descriptive results

Regarding education, 39.7% of men and 43.5% of women had lower secondary education or less, 26.6% of men and 38.8% of women had basic upper secondary education, 21.9% of men and 7.7% of women had final upper secondary education, and 11.8% of men and 10.0% of women had higher education. The average income for husbands was NOK 90,835 (*sd* = 68,404) and for wives 38,170 (*sd* = 41,150).

The follow-up time averaged 15.5 years for the respondents. During this time, 1,454 (7.3%) couples divorced or separated, while 5,732 (28.9%) of the observations became censored before the end of the study because either of the spouses died. This corresponds to a divorce rate of 5.3 per 1,000 living couples per year. On average, the couples had been married for 25.0 years when the study took place. Couples that later divorced had on average been married for 12.3 years when the study took place, and divorced on average 7.1 years after the study, which means that the average divorce in this study took place after 19.5 years of marriage. Among the couples aged 20–29 at baseline, 23.4% divorced, whereas only 0.4% of couples aged 60 years of older divorced.

The distribution of the predictor-variables are shown in Table 1. Prevalences of the health indicators ranged from 36.2% from smoking among husbands to 2.6% for heavy drinking among wives. There were significant spouse resemblances for all the predictors. Interspouse correlations ranged from 0.20 for obesity to 0.96 for age. All the interspouse correlations are shown in Table 2.

**Table 1:** **Prevalence of the different health characteristics among husbands and wives**

	**Husbands**	**Wives**
Poor subjective health	26.2%	27.0%
Obesity	7.8%	13.7%
Heavy drinking	11.6%	2.6%
Mental distress	6.4%	10.6%
No exercise	40.4%	39.6%
Smoking	36.2%	30.1%

**Table 2:** **Spousal similarity** (**between-partner correlations) for each characteristic**

**Characteristic**	**r**
Poor subjective health	.44
Obesity	.20
Heavy drinking	.48
Mental distress	.30
No exercise	.39
Smoking	.48
Age	.96
Income	.36
Education	.49

Note: p < 0.001 for all correlations. Tetrachoric correlations calculated for the six health variables, Pearson correlations for age, income and education.

### Survival analysis

The results of the survival analyses are shown in Table 3. In the left column, the husbands’ and wives’ scores for each health indicator are entered into the model together with the interaction term. These results are adjusted for age, income, education, interactions between the spouses on these variables, and the length of marriage. The right column shows the results when all predictors are entered into the model simultaneously.

**Table 3:** **Hazard ratios (HR) of marital dissolution associated with six health and health behavior variables**

	**Partially adjusted** ^**b**^				**Fully adjusted** ^**c**^			
	**HR**	**95%**	**C.I.**	**p**	**HR**	**95%**	**C.I.**	**p**
***Poor subjective health***								
None	1.00				1.00			
Husband only	1.30	1.10	1.54	0.002	1.11	0.93	1.32	0.259
Wife only	1.37	1.16	1.62	<0.001	1.06	0.89	1.26	0.530
Interaction	0.96	0.70	1.33	0.819	0.99	0.71	1.37	0.930
Both (product)^a^	1.72	1.35	2.20	<0.001	1.15	0.89	1.50	0.284
***Obesity***								
None	1.00				1.00			
Husband only	0.93	0.72	1.21	0.595	0.97	0.74	1.25	0.791
Wife only	1.08	0.87	1.35	0.480	1.11	0.89	1.38	0.363
Interaction	0.84	0.41	1.69	0.622	0.86	0.42	1.74	0.671
Both (product)^a^	0.84	0.45	1.58	0.596	0.92	0.49	1.72	0.788
***Heavy drinking***								
None	1.00				1.00			
Husband only	1.49	1.30	1.70	<0.001	1.29	1.13	1.48	<0.001
Wife only	2.00	1.51	2.66	<0.001	1.62	1.22	2.16	0.001
Interaction	0.58	0.38	0.90	0.014	0.65	0.42	1.00	0.049
Both (product)^a^	1.73	1.26	2.36	0.001	1.35	0.99	1.86	0.060
***Mental distress***								
None	1.00				1.00			
Husband only	2.06	1.69	2.51	<0.001	1.89	1.54	2.33	<0.001
Wife only	2.51	2.15	2.93	<0.001	2.26	1.92	2.67	<0.001
Interaction	0.64	0.43	0.97	0.034	0.64	0.42	0.97	0.034
Both (product)^a^	3.32	2.38	4.63	<0.001	2.74	1.93	3.89	<0.001
***No exercise***								
None	1.00				1.00			
Husband only	1.15	1.00	1.33	0.047	1.02	0.88	1.17	0.824
Wife only	1.18	1.02	1.37	0.029	1.07	0.92	1.24	0.385
Interaction	0.73	0.59	0.90	0.004	0.78	0.63	0.96	0.019
Both (product)^a^	0.99	0.86	1.14	0.884	0.84	0.73	0.97	0.017
***Smoking***								
None	1.00				1.00			
Husband only	1.78	1.53	2.08	<0.001	1.71	1.46	2.00	<0.001
Wife only	1.64	1.40	1.92	<0.001	1.53	1.30	1.79	<0.001
Interaction	0.78	0.63	0.97	0.023	0.79	0.64	0.99	0.037
Both (product)^a^	2.28	1.99	2.60	<0.001	2.07	1.80	2.37	< 0.001

Notes:

^a^Effects of both spouses having a characteristic correspond to the product of main and interaction effects.

^b^The partially adjusted results are adjusted for years since marriage, age, income, education, and the spousal differences in these.

^c^All variables were entered in the model simultaneously.

#### Partially adjusted results

Five of the six health and health behavior variables were associated with a higher divorce risk, when either the husband or the wife had the characteristic. Obesity was not related to the risk of divorce. The effect sizes were of the same magnitude whether it was the husband or the wife who had the characteristic. The interaction terms were statistically significant at the 0.05 level for four of the six health indicators: heavy drinking, mental distress, no exercise and smoking. This means that the risk of divorce in couples where both spouses had a characteristic was smaller than the combined main effects associated with husbands and wives having the characteristic. Poor mental health seemed to have additive effects on the risk of divorce, as the interaction term was not significant. The interaction term for obesity was also not statistically significant. The risk of divorce in couples with two partners with a characteristic compared to none can be calculated by multiplying the main effects and the interaction effect. Couples with two spouses with poor subjective health, heavy drinking, mental distress, or smoking had higher risks of divorce than couples without these risk factors. Lack of exercise was the only characteristic associated with divorce where couples with two or no partners with the characteristic had approximately the same divorce risk.

#### Fully adjusted results

When all the predictors were entered into the regression model at the same time, the effect sizes were in general somewhat smaller than in the previous models. The main effects of husband and wife poor subjective health and no exercise were no longer statistically significant. The effects of the interaction terms were approximately the same as in the partially adjusted models, that is, four of the six interaction terms showed significant protective effects of spousal similarity. Spousal similarity in heavy drinking, mental distress, no exercise and smoking reduced the risk of divorce, compared to the combined effects.

The risk of divorce in couples with two heavy drinking spouses was not statistically significantly different from couples with no heavy drinking spouses, although it was bordering significance. Couples with two non-exercising spouses had lower risk of divorce than couples where both exercised, and couples where one of the partners exercised. For mental distress and smoking, the risk of divorce was elevated in couples concordant in these characteristics, compared to couples without these characteristics.

#### Concordant vs. discordant couples

Table 4 shows the results when discordant couples are set as the reference category. The results are similar to those presented in Table 3. For poor subjective health and obesity, the results are not statistically significant after adjusting the risk factors for each other. For heavy drinking, mental distress, and smoking, couples concordant in not having the risk factor have a significantly lower risk of divorce, compared to couples in which one spouse had the risk factor. For heavy drinking and mental distress, the risk is neither significantly increased nor decreased when both partners have the risk factor. For exercise, however, couples where neither partner exercised had a significantly reduced risk of divorce. For smoking, couples with no smokers had significantly lower, and couples with two smokers significantly higher risk of divorce, compared to couples with one smoker.

**Table 4:** **Risk of marital dissolution in concordant couples compared to discordant couples. Hazard ratios (HR) of marital dissolution**

	**HR**	**95%**	**C.I.**	**p**
***Poor subjective health***				
None	0.92	0.81	1.06	0.243
Husband or wife	1.00			
Both	1.08	0.83	1.40	0.583
***Obesity***				
None	0.95	0.80	1.13	0.579
Husband or wife	1.00			
Both	0.89	0.47	1.70	0.727
***Heavy drinking***				
None	0.75	0.66	0.85	<0.001
Husband or wife	1.00			
Both	1.03	0.74	1.42	0.875
***Mental distress***				
None	0.48	0.42	0.55	<0.001
Husband or wife	1.00			
Both	1.32	0.93	1.88	0.123
***No exercise***				
None	0.97	0.86	1.10	0.656
Husband or wife	1.00			
Both	0.81	0.71	0.93	0.002
***Smoking***				
None	0.62	0.54	0.71	<0.001
Husband or wife	1.00			
Both	1.29	1.14	1.46	<0.001

Note: The results are adjusted for years since marriage, age, income, education, and the spousal differences in these. All variables were entered in the model simultaneously.

## Discussion

This study investigated the risk of marital dissolution associated with six health characteristics and health behaviors: poor subjective health, obesity, heavy drinking, mental distress, lack of exercise, and smoking. Adjusted for demography, five of these characteristics were associated with marital dissolution (all characteristics but obesity), while spousal similarities in four of these characteristics were associated with a reduced risk of divorce (heavy drinking, mental distress, no exercise, smoking). Fully adjusted, three of these characteristics were related to marital dissolution (heavy drinking, mental distress, smoking), while spousal similarities in these characteristics and in lack of exercise were protective. Nevertheless, with the exception of lack of exercise, the effects of spousal similarities were not strong enough to outweigh the risk associated these characteristics being present in a couple.

The presence of these health issues and poor health behaviors seems to represent enduring vulnerabilities or stressors, in the terms of the VSA model, and are likely to interfere with the couples’ adaptive processes. Looking at the significant characteristics individually, heavy drinking may interfere with daily tasks and functioning (Marshal 2003). The risk associated with mental distress may reflect depression-prone individuals’ tendency to view their own marriages more negatively (Malouff et al. 2010), or that marital discord leads first to mental distress and later to marital dissolution (Amato & James 2010). While smoking can be bothersome for non-smoking partners, we believe the effects of smoking on marital dissolution are more likely to reflect the personalities of those who smoke, rather than direct effects from smoking. Smoking is more common among people high in neuroticism and low in agreeableness and conscientiousness (Terracciano & Costa 2004). These characteristics are also related to lower marital satisfaction (Malouff et al. 2010). The risk observed with lack of exercise and poor subjective health is probably best explained by other factors, as these were not significant in the fully adjusted model. For example, poor subjective health is moderately strongly correlated with neuroticism (Okun & George 1984). Only obesity did not influence the risk of divorce in any of the analyses. This contrasts a study finding that body mass influence marital satisfaction (Meltzer et al. 2011).

The interaction effects indicate that the risk associated with husbands and wives do not simply add together. In this case, there is a protective effect of spousal similarity, even spousal similarities in risk factors. None of the interaction terms were related to increased risk of marital dissolution. Accordingly, health similarity in general may be protective against divorce. The analyses of discordant and concordant couples showed that the risk of divorce was generally not increased when both partners had a risk factor, compared to when only one of them did. The risk of divorce seems to be related to the risk factor being present in the couple, rather than the number of partners having the risk factor. Unsurprisingly, the lowest risk of marital dissolution was found among couples who were similar in not having these vulnerabilities and stressors. Despite being risk factors, spousal similarities in these characteristics are likely to enhance relationship quality because similar partners react to the environment similarly (Gonzaga et al. 2007). They understand each other better and are more likely to feel validated by their partner’s similar emotional experiences. Spousal similarity in health and lifestyle factors may be related to everyday interaction, support, and the amount of time spouses spend together. Thus, concordant couples are likely to meet these health challenges with better adaptive processes than discordant couples are. This is consistent with the health mismatch hypothesis (Wilson & Waddoups 2002). Poor health could stress the couple, interfere with daily activities, or increase worrying. The effects of spousal similarity can also be interpreted within social exchange theory (Levinger 1976): Spouses with health and health behavior issues may be perceived as less attractive by their discordant partners, and may thus be at a higher risk for being selected away, whereas the difference in attractiveness is smaller in concordant couples. Couples concordant in having poor health or health behaviors may thus be more dependent on each other.

### Limitations

Although we have followed a large sample from the general population with a high response rate and with an objective outcome measure with no attrition over 15 years, our findings must be interpreted in the light of some methodological limitations. First, we did not have any measures of potentially important mediators such as attraction, the current and initial marital satisfaction, and on who initiated the divorce. Second, we cannot determine whether some of the included variables mediate others. For example, it is possible that subjective health first affect mental distress and then the risk of marital dissolution, and thus is more important than indicated by the full model. It could also be that people in happy marriages converge more than unhappy couples, rather than spousal similarity affecting the relationship quality per se. Third, the full model includes many intercorrelated variables sharing explained variance, which makes it hard to detect significant effects even in our large sample. Fourth, several of the measures were developed specifically for the HUNT study and have unknown reliability. Measurement errors will attenuate the estimated effects. Fifth, the sample came from couples in the general population married for any length of time. As some couples will have divorced prior to the inclusion in the study, the results may not be fully generalizable to newly-weds. Sixth, generalizability may be limited by time, culture, and the age of the respondents. In addition, we did not have data on same-gendered couples or on non-marital cohabiters.

### Implications

Previous studies have addressed specific health issues as risk factors for divorce (Torvik et al. 2013; Idstad et al. 2015). This study shows that spousal similarities across a range of health indicators and health behaviors are related to a reduced risk of divorce. The risk of divorce is thus likely to be related to dissimilarity in health in general, rather than dissimilarity in specific health characteristics. The health behaviors are likely to be most strongly related to everyday interaction between the spouses and time spent together. Although we cannot conclude on causal mechanisms, health behaviors may be a target in divorce prevention, with an aim of harmonizing these. Good health behaviors will also improve measures of current health, and may thus be especially efficacious targets. Knowledge of these findings may also motivate health behavior change. Future studies will benefit from combining a longitudinal design with data on likely mediators. It would also be important to study how life satisfaction develops after the divorce has taken place, i.e. whether divorcing was a good or bad choice, depending on similarity.

## Conclusion

The present study demonstrates that health, health behaviors, and differences between the partners in these all contribute independently to the risk of divorce. Five out of six health variables indicated a higher risk of divorce, and may be seen as vulnerabilities or stressors affecting the adaptive processes of a couple. Spousal similarities in four of the six characteristic were related to marital stability, although presence of these characteristics still represented a risk. Similarity in health thus seems to be important for marital stability. This study demonstrates that people who are similar to each other are more likely to stay together. Health behaviors may be a target in divorce prevention.
